# Stability of the HTLV-1 Antisense-Derived Protein, HBZ, Is Regulated by the E3 Ubiquitin-Protein Ligase, UBR5

**DOI:** 10.3389/fmicb.2018.00080

**Published:** 2018-01-30

**Authors:** Amanda R. Panfil, Jacob Al-Saleem, Cory M. Howard, Nikoloz Shkriabai, Mamuka Kvaratskhelia, Patrick L. Green

**Affiliations:** ^1^Department of Veterinary Biosciences, Center for Retrovirus Research, The Ohio State University, Columbus, OH, United States; ^2^Division of Infectious Diseases, School of Medicine, University of Colorado Denver, Aurora, CO, United States; ^3^Comprehensive Cancer Center and Solove Research Institute, The Ohio State University, Columbus, OH, United States

**Keywords:** HTLV-1, HBZ, UBR5, ubiquitin, proliferation, T-cell

## Abstract

Human T-cell leukemia virus type 1 (HTLV-1) encodes a protein derived from the antisense strand of the proviral genome designated HBZ (HTLV-1 basic leucine zipper factor). HBZ is the only viral gene consistently expressed in infected patients and adult T-cell leukemia/lymphoma (ATL) tumor cell lines. It functions to antagonize many activities of the Tax viral transcriptional activator, suppresses apoptosis, and supports proliferation of ATL cells. Factors that regulate the stability of HBZ are thus important to the pathophysiology of ATL development. Using affinity-tagged protein and shotgun proteomics, we identified UBR5 as a novel HBZ-binding partner. UBR5 is an E3 ubiquitin-protein ligase that functions as a key regulator of the ubiquitin proteasome system in both cancer and developmental biology. Herein, we investigated the role of UBR5 in HTLV-1-mediated T-cell transformation and leukemia/lymphoma development. The UBR5/HBZ interaction was verified *in vivo* using over-expression constructs, as well as endogenously in T-cells. shRNA-mediated knockdown of UBR5 enhanced HBZ steady-state levels by stabilizing the HBZ protein. Interestingly, the related HTLV-2 antisense-derived protein, APH-2, also interacted with UBR5 *in vivo*. However, knockdown of UBR5 did not affect APH-2 protein stability. Co-immunoprecipitation assays identified ubiquitination of HBZ and knockdown of UBR5 resulted in a decrease in HBZ ubiquitination. MS/MS analysis identified seven ubiquitinated lysines in HBZ. Interestingly, UBR5 expression was upregulated in established T lymphocytic leukemia/lymphoma cell lines and the later stage of T-cell transformation *in vitro*. Finally, we demonstrated loss of UBR5 decreased cellular proliferation in transformed T-cell lines. Overall, our study provides evidence for UBR5 as a host cell E3 ubiquitin-protein ligase responsible for regulating HBZ protein stability. Additionally, our data suggests UBR5 plays an important role in maintaining the proliferative phenotype of transformed T-cell lines.

## Introduction

Categorized as a tumorigenic virus, human T-cell leukemia virus type 1 (HTLV-1) is a deltaretrovirus that infects and transforms CD4+ T-cells. Approximately 10–15 million people worldwide are infected, with areas of endemic infection in southwestern Japan, Africa, South America, and the Pacific Islands (DeThe and Bomford, 1993; Matsuoka and Jeang, 2005; Gessain and Cassar, 2012). HTLV-1 is responsible for a highly aggressive and chemotherapy-resistant peripheral T-cell malignancy called adult T-cell leukemia/lymphoma (ATL) (Uchiyama et al., 1977; Poiesz et al., 1980; Yoshida et al., 1982) and a chronic progressive neurodegenerative disease termed HTLV-1-associated myelopathy/tropical spastic paraparesis (HAM/TSP) (Gessain et al., 1985; Osame et al., 1986). As a blood borne pathogen, HTLV-1 is primarily spread through breastfeeding, blood transfusions, and sexual contact. Not all HTLV-1 carriers will go on to develop disease. Disease penetrance is roughly 5% for the lifetime of an infected individual (Ishitsuka and Tamura, 2014). The discrepancy between individuals with disease development and lifelong healthy carriers is still not well understood.

Studies have identified at least two viral genes, *tax* and *hbz*, that are linked to oncogenic transformation and involved in the pathogenic process (Cheng et al., 2012; Ma et al., 2016). Derived from the sense strand of the proviral genome, Tax serves as a transcriptional activator of both viral and cellular gene expression (Bex and Gaynor, 1998). In addition, it deregulates the cell cycle, which ultimately leads to the accumulation of cellular genetic mutations (Marriott and Semmes, 2005). Although essential for viral transformation, Tax is frequently absent from ATL tumor cells through epigenetic silencing, 5′ LTR deletion, or abortive protein mutations in the *tax* gene (Takeda et al., 2004).

Conversely, a viral transcript that is consistently found in ATL cells is the antisense-derived *hbz* transcript (Satou et al., 2006). *Hbz* transcription initiates in the mostly epigenetically unmodified 3′ LTR (Larocca et al., 1989; Satou et al., 2006). Viral cAMP-responsive elements (CRE) and several SP1 binding sites help regulate transcription of *hbz* (Yoshida et al., 2008). *Hbz* mRNA exists in both a spliced and unspliced transcript variant (Satou et al., 2006). The proteins encoded by these transcripts have nearly identical amino acid sequence (with the exception of the first several amino acids) and demonstrate several functional differences in cells (Yoshida et al., 2008). Spliced HBZ is more abundant in infected cells (Usui et al., 2008) and therefore most research to date has focused on this isoform. The spliced *hbz* transcript encodes a 206-amino acid nuclear protein comprised of 3 domains: an N-terminal activation domain, a central basic region, and a C-terminal bZIP domain (Gaudray et al., 2002; Zhao and Matsuoka, 2012). Within the activation domain are two well-characterized LXXLL-like motifs. These motifs have been shown to bind the KIX domain of CBP/p300 and are also required for HBZ to activate TGF-β signaling (Clerc et al., 2008; Zhao et al., 2011). Through its bZIP domain, HBZ is able to hetero-dimerize with cellular bZIP proteins and affect their binding to DNA recognition sites (Matsuoka and Green, 2009).

Deletion of HBZ expression in the context of the virus has been studied using an HTLV-1 infectious molecular clone with a premature stop codon in HBZ, termed HTLV-1 ΔHBZ (Arnold et al., 2006). HBZ knockout had little effect on viral infectivity and transformation of T-cells in cellular immortalization assays *in vitro*. However, when the HTLV-1 ΔHBZ virus was injected in rabbits, there was a significant decrease in antibody response to the virus and proviral load, suggesting a positive role of HBZ in viral infectivity or proliferation of infected cells *in vivo*. In monkeys, the HBZ-KO clone gradually reverts to wild-type during prolonged infection (Valeri et al., 2010), confirming the importance of HBZ in viral infectivity and persistence *in vivo*.

A popular theory within the HTLV-1 field has been that Tax is responsible for initiating transformation, while HBZ provides the maintenance or cell survival signals later during transformation. However, HBZ expression is found both in early and late stages of viral infection (Li et al., 2009). In addition, it has been shown that HBZ is able to sustain proliferative cellular signaling, evade growth suppressors, resist cell death, cause genomic instability and mutations, enable replicative immortality, induce tumor-promoting inflammation, and has low immunogenicity (Ma et al., 2016). Therefore, the view is shifting to appreciate the role of HBZ throughout HTLV-1-mediated oncogenesis.

To date, little is known regarding the regulation of HBZ protein expression. We therefore sought to identify cellular proteins that regulate HBZ stability using affinity capture coupled with shotgun proteomics. These experiments have identified UBR5 as a novel HBZ-interacting partner. UBR5 is an E3 ubiquitin-protein ligase that functions as a key regulator of the ubiquitin proteasome system in both cancer and developmental biology. Through shRNA-mediated knockdowns and cycloheximide pulse chase experiments, we found that UBR5 regulates HBZ protein stability. MS/MS analysis enabled us to discover ubiquitination at seven lysine residues throughout the HBZ protein. Interestingly, UBR5 protein is over-expressed in T-cell lymphoma cell lines and using our HTLV-1-mediated model of T-cell transformation, we determined this dysregulation occurs late during the transformation process. In addition, we found UBR5 positively regulates cellular proliferation in T-cell lymphoma cell lines. Taken together, our data identifies UBR5 as an E3 ubiquitin-protein ligase responsible for the regulation of HBZ protein stability. In addition, it suggests UBR5 may play a role in maintaining the proliferative phenotype of transformed T-cell lines.

## Materials and Methods

### Cell Lines and Culture

HEK293T cells were maintained in Dulbecco’s modified Eagle’s medium (DMEM) supplemented with 10% fetal bovine serum (FBS) (Gemini Bio-Products, Broderick, CA, United States), 2 mM glutamine, penicillin (100 U/mL), and streptomycin (100 μg/mL). PBL-ACH (early passage HTLV-1-immortalized human T-cells) were maintained in RPMI 1640 supplemented with 20% FBS, 20 U/mL recombinant human interleukin-2 (rhIL-2; Roche Applied Biosciences, Indianapolis, IN, United States), 2 mM glutamine, penicillin (100 U/mL), and streptomycin (100 μg/mL). SLB-1 cells (HTLV-1-transformed T-cell line) were maintained in Iscove’s medium supplemented with 10% FBS, 2 mM glutamine, penicillin (100 U/mL), and streptomycin (100 μg/mL). C8166, MT-1, MT-2, Hut-102 (HTLV-1-transformed T-cell lines), Hut-78, Jurkat (HTLV-1-negative transformed T-cell lines), TL-Om1, ATL-43T, and ATL-ED cells (ATL-derived T-cell lines) were maintained in RPMI 1640 supplemented with 10% FBS, 2 mM glutamine, penicillin (100 U/mL), and streptomycin (100 μg/mL). ATL-55T (ATL-derived T-cell line) were maintained in RPMI 1640 supplemented with 10% FBS, 20 U/mL rhIL-2, 2 mM glutamine, penicillin (100 U/mL), and streptomycin (100 μg/mL). The parental 729.B (uninfected) and derivative 729.ACH (HTLV-1 producing) cell lines were maintained in Iscove’s medium supplemented with 10% FBS, 2 mM glutamine, penicillin (100 U/mL), and streptomycin (100 μg/mL). All cells were grown at 37°C in a humidified atmosphere of 5% CO_2_ and air. Human PBMCs were isolated using Ficoll-Paque PLUS (GE Healthcare Life Sciences, Pittsburgh, PA, United States) and naïve T-cells were enriched using a Pan T-Cell Isolation Kit (Miltenyi Biotec., Inc., Gaithersburg, MD, United States), while memory CD4 T-cells were isolated using a Memory CD4+ T Cell Isolation Kit (Miltenyi Biotec, Inc.)

### Plasmids and Cloning

Plasmid DNA was purified on maxi-prep columns according to the manufacturer’s protocol (Qiagen, Valencia, CA, United States). The S-tagged APH-2 and HBZ expression vectors and the pME-APH-2 and HBZ expression vectors were generated and described previously (Arnold et al., 2006; Yin et al., 2012; Panfil et al., 2016). pCMV-Tag2B-EDD (Flag-UBR5) was a gift from Darren Saunders & Charles Watts (Addgene plasmid #37188) (Henderson et al., 2002). Flag-tagged ubiquitin was provided by F. Nina Papavasiliou (The Rockefeller University, New York, NY, United States) (Delker et al., 2013; Chesarino et al., 2014). S-tagged-BR, bZIP, and BR/bZIP were cloned by PCR amplifying the respective fragments from pME-HBZ and inserting them into pTriEx^TM^-4 Neo (Millipore Sigma, Burlington, MA, United States) using BamHI and EcoRI restriction sites. HBZ plasmids S-tagged-AD, AD-BR2, AD-BR2-BR1, AD-BR2-BR1-BR3, and ΔbZIP were constructed using PCR-based site-directed mutagenesis to create stop codons with the following primer sets: HBZ AD forward (5′- CGCATCGTGATCGGTAGCGACGGGCTGAGGAG -3′) and reverse (5′- CTCCTCAGCCCGTCGCTACCGATCACGATGCG -3′); HBZ AD-BR2 forward (5′- GAGCGGGAGAAATAGGAGGAAAAGCAGATTG -3′) and reverse (5′- CAATCTGCTTTTCCTCCTATTTCTCCCGCTC -3′); HBZ AD-BR2-BR1 forward (5′- GTCGCCAGGAGAAAGTAGGAAGAGCAGGAGCGC -3′) and reverse (5′- GCGCTCCTGCTCTTCCTACTTTCTCCTGGCGAC -3′); HBZ-AD-BR2-BR1-BR3 forward (5′- GCAGGAGTTGGGGTAGGATGGCTATACTAGACAGTTGG -3′) and reverse (5′- CCAACTGTCTAGTATAGCCATCCTACCCCAACTCCTGC -3′); and HBZ-ΔbZIP forward (5′- GGAAGGCGAGGTGTAGTCCTTGGAGGCTG -3′) and reverse (5′- CAGCCTCCAAGGACTACACCTCGCCTTCC -3′). HBZ cDNA was cloned into the pCDH-EF1-MCS-T2A-copGFP lentiviral expression vector (SBI, Mountain View, CA, United States) to create an HBZ-GFP chimeric protein and used to generate the Jurkat-HBZ cell line.

### S-Tag Affinity Pulldown Assays

HEK293T cells were transfected with the indicated expression vectors using Lipofectamine^®^2000 (Life Technologies, Carlsbad, CA, United States) according to the manufacturer’s instructions. Twenty-four hours post-transfection, cells were treated with 10 μM MG-132 (Sigma–Aldrich, St. Louis, MO, United States) for 20 h. Cells were washed with 1× PBS and lysates were prepared with NP-40 lysis buffer in the presence of protease inhibitor (Roche Applied Biosciences) and 25 mM *N*-Ethylmaleimide (Sigma–Aldrich). Cells were centrifuged at maximum speed for 10 min at 4°C. S-tag purification was performed by rocking cell lysates with S beads (Millipore Sigma) overnight at 4°C. The S beads were washed twice with NP-40 lysis buffer. An equal volume of 2× SDS-sample buffer was added and proteins were extracted by heating at 95°C for 10 min.

### Mass Spectrometry and Proteomic Analysis

The pulled-down proteins were subjected to SDS-PAGE and visualized by GelCode Blue staining. For identification of cellular binding partners of S-tagged HBZ and S-tagged APH-2 entire lanes were excised and cut into small pieces. For identification of ubiquitination sites the prominent S-tagged HBZ bands were excised. The gel pieces were then de-stained with 50% acetonitrile and then subjected to in-gel proteolysis using sequencing grade modified trypsin (Promega, Madison, WI, United States). The resulting peptides were extracted in acetonitrile by vortexing for 10 min and then desiccated in Eppendorf^®^ Vacufuge^®^ Plus Vacuum Concentrator. The samples were run on Thermo Scientific Q Exactive mass spectrometer and analyzed with Mascot software (Matrix Science, Boston, MA, United States). The data were visualized using Scaffold Viewer (Proteome Software, Inc., Portland, OR, United States) using 1.0% FDR Protein Threshold, 2 Min. #Peptides, and 1.0% Peptide Threshold.

### Flag Immunoprecipitation Assays

HEK293T cells were transfected with the indicated expression vectors using Lipofectamine^®^2000 (Life Technologies) according to the manufacturer’s instructions. Twenty-four hours post-transfection, cells were treated with 10 μM MG-132 (Sigma–Aldrich) for 20 h. Cells were washed with 1× PBS and then incubated with gentle rocking at 4°C for 30 min in 0.5% NP-40 lysis buffer (150 mM NaCl, 0.5% NP-40, 50 mM Tris-HCl, pH 8.0) in the presence of protease inhibitor (Roche Applied Biosciences) and 25 mM NEM (Sigma–Aldrich). Cells were centrifuged at maximum speed for 10 min at 4°C. The lysates were then diluted to 0.1% NP-40 lysis buffer. Flag immunoprecipitation was performed by rocking cell lysates with ANTI-FLAG^®^ M2 Affinity Gel (Sigma–Aldrich) overnight at 4°C. The FLAG resin was washed twice with 0.1% NP-40 lysis buffer. An equal volume of 2× SDS-sample buffer was added and proteins were extracted by heating at 95°C for 10 min.

### Immunoprecipitation Assays

Lymphocytes were treated with 10 μM MG-132 (Sigma–Aldrich) for 20 h. Cells were washed with 1× PBS and then incubated with gentle rocking at 4°C for 30 min in NP-40 lysis buffer in the presence of protease inhibitor (Roche Applied Biosciences) and 25 mM NEM (Sigma–Aldrich). Cells were centrifuged at maximum speed for 10 min at 4°C. Antibody (1 to 2 μg or no antibody for the direct load) was added to each sample, and the samples were rocked overnight at 4°C. The antibodies used were as follows: control rabbit IgG (Santa Cruz Biotechnology, Dallas, TX, United States), rabbit anti-HBZ antiserum, and rabbit anti-UBR5 (Bethyl Laboratories, Inc., Montgomery, TX, United States). Protein G Dynabeads (Fisher Scientific, Hampton, NH, United States) were added and the mixture was rocked at 4°C for 2 h. The beads were washed twice in NP-40 lysis buffer. An equal volume of 2× SDS-sample buffer was added and proteins were extracted by heating at 95°C for 10 min.

### Immunoblotting

Cell lysates were harvested in NP-40 lysis buffer containing protease inhibitor cocktail (Roche Applied Bioscience) and quantitated using a Pierce bicinchoninic acid protein assay kit (Fisher Scientific). Equivalent amounts of protein were separated in Mini-Protean TGX precast 4 to 20% gels (Bio-Rad Laboratories, Hercules, CA, United States) and transferred to nitrocellulose membranes. Membranes were blocked in phosphate-buffered saline (PBS) containing 5% milk and 0.1% Tween 20 and incubated with primary antibody. The following antibodies were used: anti-S-tag (1:1000; Abcam, Cambridge, MA), anti-UBR5 (1:1000; Cell Signaling Technology, Danvers, MA, United States), anti-HBZ (1:1,000), anti-APH-2 (1:1,000), anti-FLAG clone M2 (1:1,000; Agilent Technologies, Santa Clara, CA, United States), anti-ubiquitin (1:250; Santa Cruz Biotechnology), anti-β-actin (1:5,000; Sigma–Aldrich), and anti-α-Tubulin (1:250; Santa Cruz Biotechnology). The secondary antibodies used were horseradish peroxidase-labeled goat anti-rabbit and goat anti-mouse immunoglobulin antibodies (1:5,000; Santa Cruz Biotechnology). The blots were developed using an ECL Western Blotting Substrate (Fisher Scientific). Images were taken using an Amersham Imager 600 imaging system (GE Healthcare Life Sciences), and densitometric data were calculated using the ImageQuant TL program (GE Healthcare Life Sciences).

### Co-culture Immortalization Assays

Long-term immortalization assays were performed as detailed previously (Green et al., 1995). Briefly, 2 × 10^6^ freshly isolated human PBMCs were co-cultivated at a 2:1 ratio with lethally irradiated cells (729.B uninfected parental; 729.ACH HTLV-1-producing) in 24-well culture plates (media was supplemented with 10 U/mL rhIL-2).

### Quantitative RT-PCR

Total RNA was isolated using an RNeasy Mini kit (Qiagen) according to the manufacturer’s instructions. Isolated total RNA was quantitated and DNase-treated using recombinant DNase I (Roche Applied Biosciences). Reverse transcription (RT) was performed using a SuperScript first-strand synthesis system for RT-PCR (Life Technologies) according to the manufacturer’s instructions. The instrumentation and general principles of the CFX96 Touch real-time PCR detection system (Bio-Rad Laboratories) are described in detail in the operator’s manual. PCR amplification was carried out in 96-well plates with optical caps. The final reaction volume was 20 μl and consisted of 10 μl iQ SYBR green Supermix (Bio-Rad Laboratories), 300 nM each specific primer, and 1.5 μl of cDNA template. For each run, standard cDNA, sample cDNA, and a no-template control were all assayed in triplicate. The reaction conditions were 95°C for 5 min, followed by 40 cycles of 94°C for 30 s, 56°C for 30 s, and 72°C for 45 s. Primer pairs for the specific detection of *ubr5* and human glyceraldehyde-3-phosphate dehydrogenase (*hgapdh*) were described previously (Subbaiah et al., 2016). Data from triplicate experiments are presented in histogram form as means with standard deviations. The total *ubr5* copy number for each cell line was determined using a plasmid DNA standard curve and normalized to 10^6^ copies of hGAPDH mRNA.

### Cycloheximide Pulse-Chase Experiments

HEK293T cells were transiently transfected with empty or untagged (pME) HBZ or APH-2 expression vectors using Lipofectamine^®^2000 (Life Technologies) according to the manufacturer’s instructions. Forty-eight hours later, the cells were treated with 100 μg/ml cycloheximide (a translation elongation inhibitor; Sigma–Aldrich) and then harvested at different time points. Jurkat-HBZ cells were synchronized by serum starvation in 0.1% FBS overnight prior to treatment with 100 μg/ml cycloheximide and then harvested at different time points.

### Infection and Packaging of Lentiviral Vectors

Lentiviral vectors expressing five different UBR5-directed short hairpin RNAs (shRNAs) (target set RHS4533-EG51366) and the universal negative control pLKO.1 (RHS4080) were purchased from Open Biosystems (Fisher Scientific) and propagated according to the manufacturer’s instructions. HEK293T cells were transfected with lentiviral vector(s) plus DNA vectors encoding HIV Gag/Pol and vesicular stomatitis virus G in 10-cm dishes using Lipofetamine^®^2000 reagent according to the manufacturer’s instructions. Media containing the lentiviral particles were collected 72 h later and filtered through 0.45-μm-pore-size filters (Fisher Scientific). Lentiviral particles were then concentrated using ultracentrifugation in a Sorvall SW-41 swinging bucket rotor at 90,000 × *g* for 1.5 h at 4°C. Target cells were infected with the indicated lentivirus by spininoculation at 2,000 × *g* for 2 h at room temperature. Three-days post-transduction, the cells were selected with puromycin for 7–10 days.

### Proliferation Assays

Cell Titer 96 Aqueous One Solution Cell Proliferation Assays (Promega) were performed according to the manufacturer’s protocol. Briefly, cells were counted and plated at 1,000 cells/well in 96-well round-bottom plates on day 0 and monitored over a 7-day time course. Cell Titer 96 reagent was added to each well, agitated slightly, and incubated at 37°C, 5% CO_2_ for 2 h. The optical density absorbance at 490 nm was collected on an enzyme-linked immunosorbent assay (ELISA) plate reader. For each cell line, data represent three independent experiments performed in triplicate.

### Annexin V Staining

Cells were stained using the FITC Annexin V Apoptosis Detection Kit I (BD Biosciences, San Jose, CA, United States) according to the manufacturer’s instructions. Cells were analyzed for apoptosis via flow cytometry using a Guava EasyCyte Mini machine (Millipore Sigma).

## Results

### HBZ Interacts with the Cellular E3 Ubiquitin-Protein Ligase, UBR5

**FIGURE 1:**
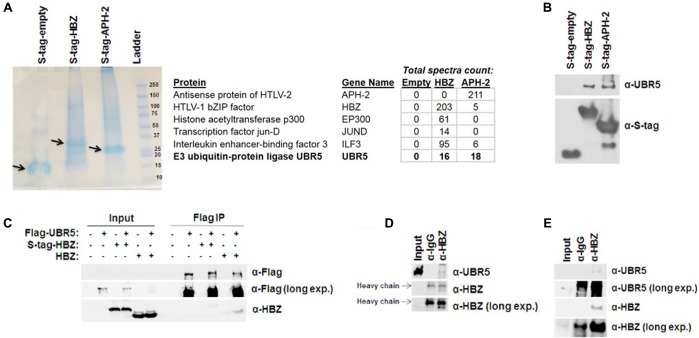
HBZ interacts with the cellular E3 ubiquitin-protein ligase, UBR5. **(A)** (Left) S-tagged HBZ and APH-2 were purified from lysates of transfected HEK293T cells using S beads. The purified APH-2 or HBZ and associated protein complexes were resolved by SDS-PAGE and visualized with GelCode Blue staining prior to MS/MS analysis. Arrow indicates enriched S-tagged protein. (Right) Brief summary of HBZ and APH-2 interacting proteins identified by MS/MS. **(B)** HEK293T cells were transfected with empty, HBZ, or APH-2 S-tagged vectors. Tagged proteins were purified by S-tag affinity purification 48 h after transfection. Pulldowns were examined by immunoblot analysis using anti-S-tag and anti-UBR5 antibodies, as indicated. **(C)** HEK293T cells were transfected with FLAG-tagged UBR5 and empty, S-tagged HBZ or untagged HBZ expression vectors, as specified. FLAG IPs were performed 48 h after transfection as described in the Section “Materials and Methods.” Immunoprecipitated proteins were examined by immunoblot analysis using anti-FLAG antibody and anti-HBZ antisera, as indicated. Five percent of the direct load was used for immunoblot analysis. **(D)** Jurkat-HBZ and **(E)** SLB-1 (HTLV-1-transformed; Right panel) cells were immunoprecipitated with a control rabbit antibody or HBZ rabbit antisera. Immunoprecipitated proteins were then examined by immunoblot analysis using anti-UBR5 antibody or anti-HBZ antisera as indicated. Five percent of the direct load was used for immunoblot analysis.

HBZ has been linked to HTLV-1-mediated oncogenic transformation and plays a role in the pathogenic process (Ma et al., 2016). HBZ regulates several cellular functions and signaling pathways (NF-κB, CBP/p300, TGF-β/Smad, CREB2, c-Jun, JunB, JunD, CREB, MafB, ATF3, etc.) within the host cell (Matsuoka and Green, 2009). However, less is known concerning the regulation of HBZ expression. We therefore sought to identify cellular factors that interact with HBZ using affinity capture coupled with shotgun proteomics. For this, HBZ was cloned into a CMV driven pTriEx4-Neo plasmid, which created an amino terminal S-tagged HBZ expression vector. In addition, the HTLV-2 equivalent to HBZ, APH-2, was also cloned into pTriEx4-Neo. HTLV-1 and -2 are highly related retroviruses (Ciminale et al., 2014). While both viruses transform T-cells *in vitro* (Shoji et al., 2009; Imai et al., 2013; Romanelli et al., 2013), they have distinct pathological outcomes *in vivo* (Arnold et al., 2006; Yin et al., 2012). Consequently, comparisons of cellular interacting partners of HBZ (HTLV-1) and APH-2 (HTLV-2) may provide a better understanding of how HTLV-1, but not HTLV-2, infection is associated with disease. S-tagged-HBZ, APH-2, or empty expression vectors were ectopically expressed in HEK293T cells. Proteins were purified using S-tag affinity pull down assays. Samples were then run on a denaturing SDS-PAGE gel (**Figure 1A**). Entire lanes were subjected to trypsin digestion and analyzed by MS/MS. An abbreviated summary of the identified interacting cellular partners is depicted (**Figure 1A**). Several known cellular binding partners of both HBZ and APH-2 were identified, as well as the novel E3 ubiquitin-protein ligase UBR5. The interaction of HBZ and APH-2 with UBR5 was confirmed using S-tag affinity pulldowns in HEK293T cells followed by immunoblot for endogenous UBR5 (**Figure 1B**). Similar results were obtained using reciprocal coIPs in HEK293T cells with Flag-tagged UBR5 and either S-tagged or untagged HBZ expression constructs (**Figure 1C**). The interaction between UBR5 and HBZ was also confirmed in the more physiologically relevant cell lines Jurkat and SLB-1 (**Figures 1D,E**). Jurkat-HBZ cells are a transformed HTLV-1-negative T-cell line that ectopically expresses HBZ (see section “Materials and Methods”). SLB-1 cells are an HTLV-1-transformed T-cell line and therefore express physiologically relevant levels of HBZ protein. Taken together, these results identify and confirm UBR5 as a novel cellular binding partner of HBZ.

### UBR5 Regulates HBZ Protein Stability

**FIGURE 2:**
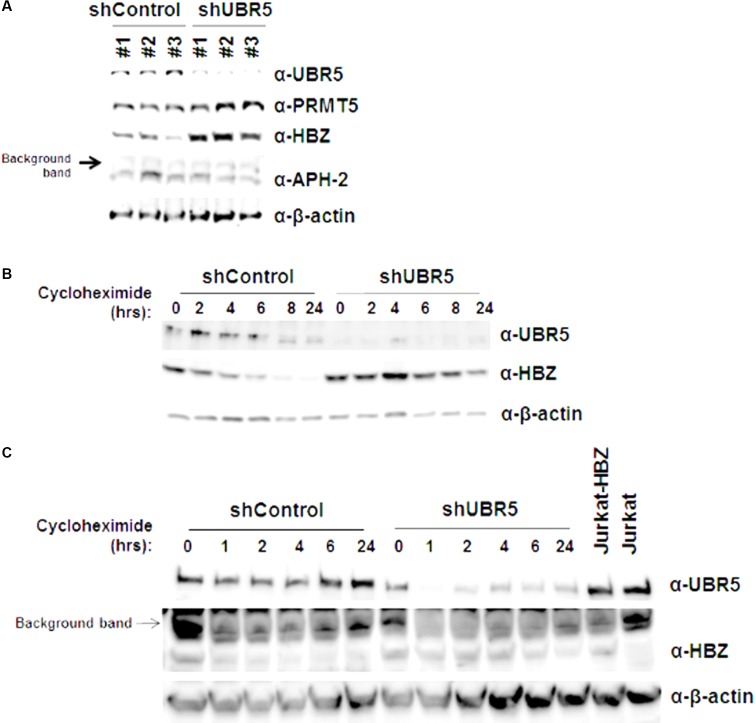
UBR5 regulates HBZ protein stability. **(A)** HEK293T cells were infected with three different lentiviral vectors expressing shRNA directed against UBR5, or control shRNAs. After a brief puromycin selection, the cells were then transfected with HBZ or APH-2 expression plasmid. After 48 h, immunoblot analysis was performed to detect HBZ, APH-2, and UBR5 expression levels. β-actin was used as a loading control while PRMT5 was used as an internal control. **(B)** HEK293T and **(C)** Jurkat-HBZ cells were infected with either a lentiviral vector directed against UBR5 or a control shRNA. After a brief puromycin selection, the HEK293T cells were transfected with HBZ expression plasmid and used for cycloheximide pulse chase experiments after 48 h. Cells were treated with 100 μg/ml cycloheximide for the indicated times. Immunoblot analysis was performed to detect HBZ, UBR5, and β-actin (loading control) expression levels.

UBR5 is a cellular E3 ubiquitin-protein ligase involved in targeting proteins for ubiquitin-mediated proteolysis. We therefore examined the steady-state levels of both HBZ and APH-2 in the presence and with reduced amounts of endogenous UBR5. HEK293T cells were transduced with three separate shUBR5 or control lentiviral vectors. After a brief selection in puromycin (7–10 days), the cells were transfected with either HBZ or APH-2 expression vectors. When the level of UBR5 is decreased, the steady-state levels of HBZ, but not APH-2, are increased compared to control infected cells (**Figure 2A**). Given the disparity between HBZ expression levels in the presence and absence of UBR5, we next utilized cycloheximide pulse chase experiments to examine HBZ protein half-life. shControl and shUBR5 HEK293T cells were transfected with an untagged HBZ expression construct and then treated with the translation elongation inhibitor cycloheximide. Cell lysates were collected at defined time points, and the expression level of HBZ was examined by immunoblot analysis (**Figure 2B**). The calculated half-life of HBZ in shControl cells was approximately 6.7 h, consistent with previous reports (Panfil et al., 2016), whereas the calculated half-life in shUBR5 cells was greater than 24 h. As expected, based on the results in **Figure 2A**, the loss of UBR5 had no effect on the half-life of APH-2 in HEK293T cells (data not shown). The half-life of HBZ was also measured in Jurkat-HBZ cells transduced with either shControl or shUBR5 lentiviral vectors (**Figure 2C**). The calculated half-life of HBZ in Jurkat-HBZ shControl cells was 2.7 h and this increased roughly twofold to 5.8 h in Jurkat-HBZ shUBR5 cells. These results indicate that UBR5 regulates HBZ protein stability.

### UBR5 Interacts with the Central Basic Region of HBZ

**FIGURE 3:**
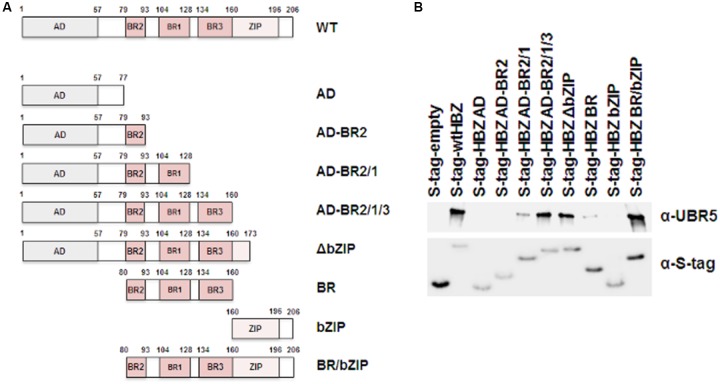
UBR5 interacts with the central basic region of HBZ. **(A)** Schematic representation of the various HBZ mutant constructs. **(B)** HEK293T cells were transfected with FLAG-tagged UBR5 and empty, wtHBZ, or HBZ mutant S-tagged expression vectors, as indicated. S-tagged proteins were purified by S-tag affinity purification 48 h after transfection. Pulldowns were examined by immunoblot analysis using anti-S-tag and anti-UBR5 antibodies, as indicated.

HBZ is a nuclear protein with an N-terminal activation domain, a central basic region, and a C-terminal basic leucine zipper domain. In order to determine the region of HBZ which interacts with UBR5, a series of mutants were created in the S-tagged HBZ expression construct as depicted in **Figure 3A**. HEK293T cells were transfected with FLAG-tagged UBR5 and either S-tagged HBZ wild-type, S-tagged HBZ mutant, or empty expression vector, as indicated. S-tag affinity pulldown assays were performed followed by immunoblot for UBR5 expression (**Figure 3B**). In addition to wild-type HBZ, the HBZ AD-BR2-BR1, HBZ AD-BR2-BR1-BR3, HBZ △bZIP, HBZ BR, and HBZ BR/bZIP mutants interacted with UBR5. These results indicate the basic region, specifically BR1 of HBZ, is important for interaction with UBR5.

### HBZ Is Ubiquitinated

**FIGURE 4:**
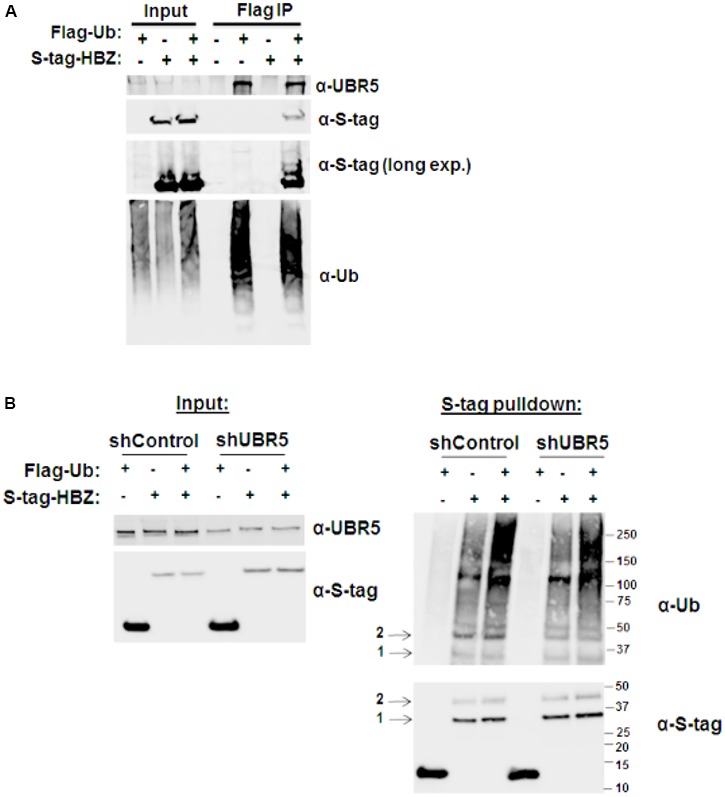
HBZ is ubiquitinated. **(A)** HEK293T cells were co-transfected with FLAG-tagged ubiquitin (UB) and/or S-tagged HBZ expression vectors. FLAG IPs were performed 48 h after transfection. Immunoprecipitated proteins were examined by immunoblot analysis using anti-ubiquitin, anti-S-tag, and anti-UBR5 antibodies as indicated. Five percent of the direct load was used for immunoblot analysis. **(B)** HEK293T cells were infected with a lentiviral vector directed against UBR5 or control lentiviral vectors. After a brief selection with puromycin, the HEK293T cells were transfected with FLAG-tagged Ubiquitin (UB) and/or S-tagged HBZ expression vectors. S-tagged proteins were purified by S-tag affinity purification 48 h after transfection. Pulldowns were examined by immunoblot analysis using anti-S-tag and anti-ubiquitin antibodies, as indicated. Five percent of the direct load was used for immunoblot analysis and probed using anti-S-tag and anti-UBR5 antibodies, as indicated. Arrow 1 indicates total HBZ, while arrow 2 indicates ubiquitinated HBZ.

As an E3 ubiquitin-protein ligase, UBR5 acts as a scaffold between its protein substrate and an E2 ubiquitin-conjugating enzyme that is loaded with ubiquitin. To determine whether UBR5 acts as an E3 ligase for HBZ, we first examined whether HBZ is ubiquitinated (**Figure 4A**). HEK293T cells were co-transfected with either FLAG-tagged ubiquitin and/or S-tagged HBZ expression constructs. FLAG co-IPs were then performed to enrich all ubiquitinated proteins. Upon ubiquitin enrichment, we observed HBZ expression by immunoblot, indicating HBZ ubiquitination. Consistent with its role as an E3 ligase, we also found UBR5 expression in the co-IP samples upon ubiquitin enrichment. Next, FLAG-tagged ubiquitin and/or S-tagged HBZ expression constructs were co-transfected into shControl and shUBR5 HEK293T cell lines. S-tag affinity pulldowns were performed to enrich total HBZ protein, followed by immunoblot for ubiquitin (**Figure 4B**). HBZ is pulled down with equal efficiency in both control and UBR5 knockdown cells as indicated by arrow 1. However, in UBR5 knockdown cells, there is less ubiquitinated HBZ present as indicated by arrow 2. Taken together, our results confirm HBZ is ubiquitinated and UBR5 serves as its E3 cellular ubiquitin-protein ligase.

### MS/MS Analysis of HBZ Ubiquitination Sites

**FIGURE 5:**
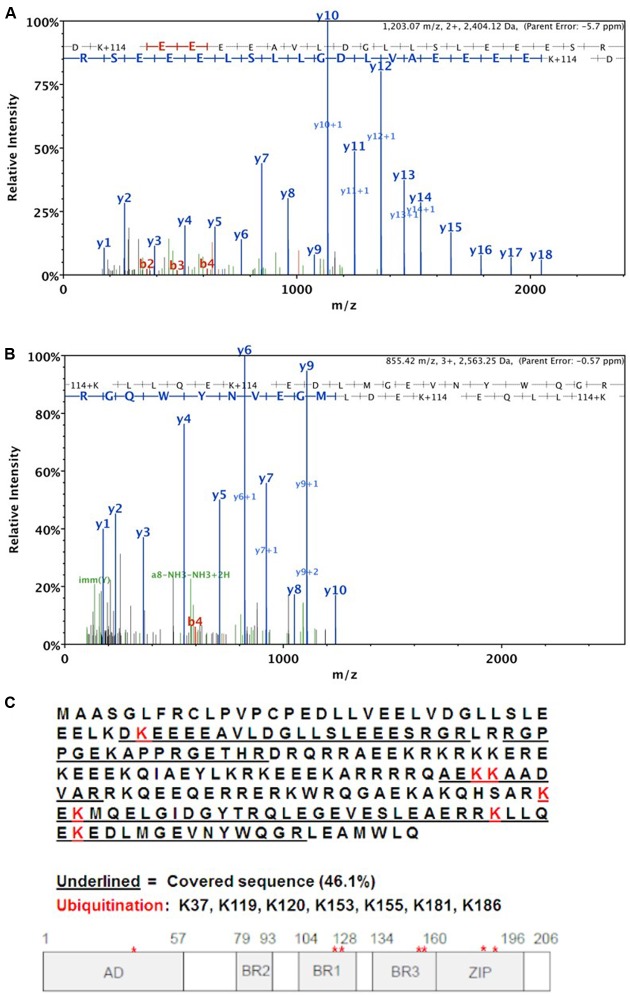
MS/MS analysis of HBZ ubiquitination sites. HEK293T cells were co-transfected with S-tagged HBZ, FLAG-UB, and FLAG-UBR5 expression vectors. S-tagged HBZ was purified from lysates of transfected HEK293T cells using S beads. The purified HBZ was resolved by SDS-PAGE prior to MS/MS analysis. Representative MS/MS fragmentation spectra are shown for the following two HBZ peptides: **(A)** DK^∗^EEEAVLDGLLSLEEES where lysine 37 is ubiquitinated. Mascot Ion score = 144.5. and **(B)** K^∗^LLQEK^∗^EDLMGEVNYWQ where lysines 181 and 186 are ubiquitinated. Mascot Ion score = 52.2. **(C)** Summary of MS results. The complete HBZ sequence is shown and the protein segments detected by MS/MS analysis are underlined. A schematic is provided to indicate ubiquitination sites on HBZ with respect to individual protein domains.

Using mass spectrometry, we analyzed the ubiquitination site(s) within the HBZ protein. HEK293T cells were transfected with FLAG-tagged ubiquitin, FLAG-tagged UBR5, and S-tagged HBZ expression constructs. S-tag affinity pulldowns were then performed to enrich all HBZ protein in the cells. The sample was then run on a denaturing SDS-PAGE gel followed by staining with GelCode Blue. The predominant HBZ bands (35, 45 kDa) were excised and digested with trypsin and subjected to MS/MS analysis, which identified seven ubiquitinated lysine residues (K37, K119, K120, K153, K155, K181, K186) in the HBZ protein (**Figure 5C**). The representative MS/MS fragmentation spectra are shown in **Figures 5A,B**, demonstrating the ubiquitination of the indicated lysines.

### UBR5 Is Dysregulated in T-Cell Leukemia/Lymphoma Cells

**FIGURE 6:**
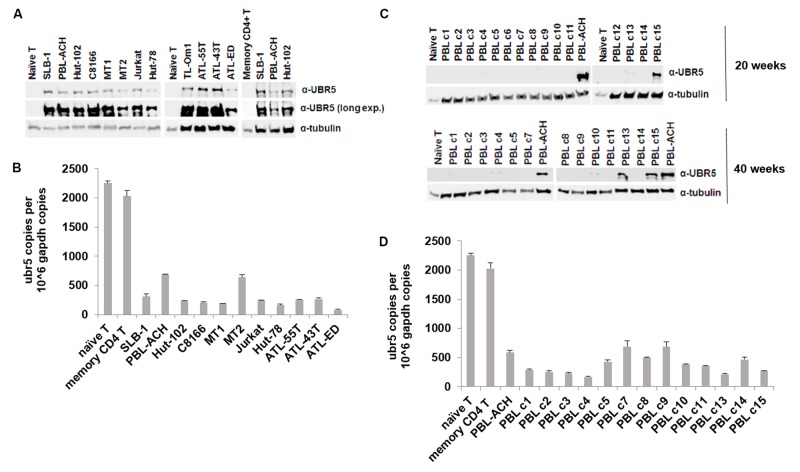
UBR5 is dysregulated in T-cell leukemia/lymphoma cells. **(A)** Total cell lysates of HTLV-1 transformed cell lines (SLB-1, PBL-ACH, Hut-102, C8166, MT-1, MT-2), HTLV-1-negative transformed cell lines (Jurkat, Hut-78), ATL-derived cell lines (TL-Om1, ATL-55T, ATL-43T, ATL-ED), and naïve and memory CD4 T-cells were subjected to immunoblot analysis to compare the levels of endogenous UBR5 expression. α-Tubulin expression was used as a loading control. **(B)** Quantitative RT-PCR for *ubr5* and *gapdh* was performed on mRNA isolated from cells in **(A)**. The total numbers of UBR5 and GAPDH copies were determined using plasmid DNA standards and normalized to 10^6^ copies of *gapdh* mRNA. **(C)** Total cell lysates of newly immortalized (20 weeks; upper panel and 40 weeks; lower panel) HTLV-1 transformed cell lines (clones 1–15), an established HTLV-1 transformed cell line (PBL-ACH) and naïve T-cells were subjected to immunoblot analysis to compare the levels of endogenous UBR5 expression. α-Tubulin expression was used as a loading control. **(D)** Quantitative RT-PCR for *ubr5* and *gapdh* was performed on mRNA isolated from cells in lower **(C)**. The total numbers of UBR5 and GAPDH copies were determined using plasmid DNA standards and normalized to 10^6^ copies of *gapdh* mRNA.

UBR5 dysregulation has been implicated in various aspects of cancer biology. UBR5 is overexpressed in both breast and ovarian cancer due to an allelic imbalance, which results in an increase in *ubr5* mRNA levels (Clancy et al., 2003). In mantle cell lymphoma, UBR5 protein is recurrently mutated (Meissner et al., 2013). To determine whether UBR5 is important to HTLV-1 biology and pathogenesis, we examined the level of UBR5 protein in a variety of transformed T-cell leukemia/lymphoma cell lines (**Figure 6A**). We included naïve primary T-cells, memory CD4 T-cells, HTLV-1-negative T-cell lines (Jurkat, Hut-78), HTLV-1-transformed T-cell lines (SLB-1, PBL-ACH, Hut-102, C8166, MT-1, MT-2), and ATL-derived T-cell lines (TL-Om1, ATL-55T, ATL-43T, ATL-ED). UBR5 protein was dramatically upregulated in all T-cell leukemia/lymphoma cell lines compared to both naïve and memory CD4 T-cells. Interestingly, UBR5 RNA levels were decreased in all T-cell leukemia/lymphoma cell lines compared to naïve and memory CD4 T-cells (**Figure 6B**), suggesting a post-transcriptional method of regulation. Using an HTLV-1-based *in vitro* T-cell immortalization co-culture assay, we determined whether UBR5 becomes dysregulated and over-expressed during the T-cell transformation process. Briefly, freshly isolated human PBMCs were co-cultured with lethally irradiated HTLV-1-producer cells. As a control, PBMCs were co-cultured with lethally irradiated HTLV-1-negative cells. Over the course of 10–14 weeks, the PBMCs co-cultured with HTLV-1-producer cells showed progressive growth indicative of HTLV-1 infection and transformation, while the PBMCs co-cultured with HTLV-1-negative cells were unable to sustain progressive growth. Using this *in vitro* transformation assay, T-cells were isolated at weekly time points and the levels of UBR5 protein were examined. We were unable to detect UBR5 protein expression at any of the time points from weeks 1–12 post-infection (data not shown) suggesting UBR5 is not upregulated early during T-cell transformation or as a result of HTLV-1 viral infection. HTLV-1-infected T-cells that became immortalized (termed HTLV-1 PBLs) continued to proliferate and grow in culture indefinitely. Several PBL clones from our *in vitro* transformation assay were kept in culture for up to 40 weeks. We examined the level of UBR5 protein expression in these cells and found upregulated UBR5 protein expression in 2 of the clones (clones 13 and 15) at 20 weeks post-infection and in 5 of the clones at 40 weeks post-infection (**Figure 6C**). Similar to T-cell leukemia/lymphoma cell lines, the level of UBR5 RNA was decreased in all PBL clones compared to naïve and memory CD4 T-cells (**Figure 6D**). These results suggest that UBR5 upregulation in T-cell lymphomas occurs late during transformation.

### UBR5 Enhances Cellular Proliferation in T-Cell Leukemia/Lymphoma Cells

**FIGURE 7:**
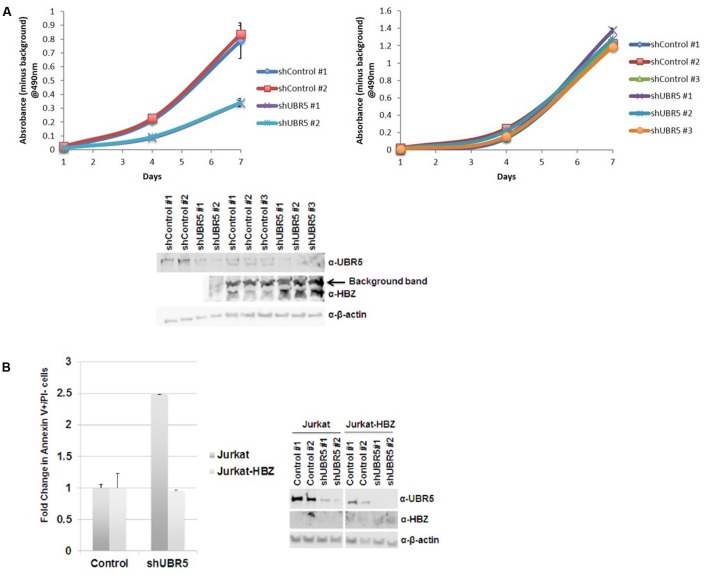
UBR5 enhances cellular proliferation in T-cell leukemia/lymphoma cells. **(A)** 10^3^ stable (Left) Jurkat and (Right) SLB-1 lentiviral control or shUBR5 infected cells were plated in normal growth medium in 96-well plates and MTS assays were performed on triplicate wells at 24-h intervals for a total of 7 days. The average absorbance numbers are plotted and error bars denote SD. (Below) Immunoblot analysis was performed on total cell lysates to compare the levels of endogenous UBR5 and HBZ expression. β-actin was used as a loading control. **(B)** Cellular apoptosis was measured using a FITC Annexin V Apoptosis Detection Kit as described in the Section “Materials and Methods.” The percentage of cells undergoing apoptosis in lentiviral control infected cells was set at 1. The fold increase in apoptosis was measured in Jurkat and Jurkat-HBZ cells infected with lentiviral shUBR5. (Right) Immunoblot analysis was performed on total cell lysates to compare the levels of endogenous UBR5 and HBZ expression. β-actin was used as a loading control.

Recently, UBR5 has been shown to regulate proliferation and colony formation of gastric cancer cells (Yang et al., 2016). To examine if UBR5 affects cellular proliferation of T-cell lymphomas, we subjected both Jurkat (HTLV-1-negative) and SLB-1 (HTLV-1-transformed) cells to shRNA-mediated knockdown of UBR5. Knockdown of UBR5 inhibited cellular proliferation of Jurkat cells, but not SLB-1 cells (**Figure 7A**). Immunoblotting revealed knockdown of UBR5 in SLB-1 cells caused an increase in HBZ steady-state levels. The level of cellular apoptosis was also measured in Jurkat and Jurkat-HBZ cells infected with shControl and shUBR5 lentivirus. The number of apoptotic cells increased 2.5-fold in shUBR5 Jurkat cells compared to the lentiviral control (**Figure 7B**). However, shUBR5 Jurkat-HBZ cells had no difference in cellular apoptosis compared to the shControl cells. These results suggest UBR5 enhances cellular proliferation in T-cell leukemia/lymphoma cell lines.

## Discussion

HTLV-1 is a tumorigenic retrovirus that preferentially infects and transforms CD4+ T-cells both *in vitro* and *in vivo*. The virus is responsible for both an aggressive T-cell neoplasm termed ATL (Uchiyama et al., 1977; Poiesz et al., 1980; Yoshida et al., 1982) and a debilitating neurodegenerative disease called HAM/TSP (Gessain et al., 1985; Osame et al., 1986). Disease development occurs in roughly 5% of infected individuals after a prolonged clinical latency period (Ishitsuka and Tamura, 2014). During this time, it is believed that both genetic and epigenetic events accumulate in the cellular environment that contributes to disease development. The virus encodes an antisense-derived protein termed HBZ, which has been shown to play a role in oncogenic transformation and proliferation (Ma et al., 2016). HBZ is the only viral gene consistently found in ATL tumors (Satou et al., 2006). While many functions of HBZ protein have been revealed, little is known regarding the regulation and stability of HBZ protein. Given the importance of HBZ during ATL development, factors that regulate the stability of HBZ are of critical value.

Using an affinity capture coupled with shotgun proteomics approach, we identified cellular factors that interact with and regulate the stability of HBZ protein. These experiments have identified a cellular E3 ubiquitin-protein ligase called UBR5 as a novel cellular interacting partner of HBZ (**Figure 1A**). UBR5 is a member of the HECT domain family of E3 ubiquitin ligases and has widespread expression in various cell types (Mansfield et al., 1994). We confirmed interactions between HBZ and UBR5 using both expression vectors in HEK293T cells and endogenous levels of HBZ/UBR5 in physiologically relevant T-cell lines, Jurkat (HTLV-1 negative) and the HTLV-1 positive, SLB-1 (**Figures 1B–E**). Using HBZ domain mutants, we identified the central basic region, specifically BR1, as essential for UBR5 interaction (**Figure 3**). This region is the site of a dominant HBZ epitope for antibody recognition (Raval et al., 2015), therefore, predominantly exposed on the HBZ protein. Of interest, UBR5 was also identified and confirmed to be an interacting partner of the closely related HTLV-2 equivalent to HBZ, called APH-2 (**Figures 1A,B**). HTLV-1 and HTLV-2 are closely related retroviruses with drastically different pathological outcomes *in vivo* (Arnold et al., 2006; Yin et al., 2012). While both viruses share many similarities, HTLV-1 is associated with disease whereas HTLV-2 has not been associated with any disease to date (Ciminale et al., 2014).

A major function of UBR5 is to target cellular proteins for ubiquitination and eventual proteasome-mediated degradation. Using shRNA-mediated lentiviral vectors, we knocked down UBR5 expression in HEK293T cells and found HBZ steady state levels were increased in response to decreased UBR5 expression (**Figure 2A**). Conversely, APH-2 steady state levels were unchanged regardless of UBR5 expression levels. As an internal control, we also examined the expression level of a cellular protein, PRMT5, and found loss of UBR5 did not result in global increases in protein expression. We previously reported the HBZ half-life to be approximately 6.4 h in HEK293T cells and 2–3 h in Jurkat cells (Panfil et al., 2016). Using shRNA-mediated UBR5 knockdown coupled with cycloheximide pulse chase experiments, we found loss of UBR5 extended the HBZ half-life to greater than 24 h in HEK293T cells and approximately 6 h in Jurkat cells (**Figures 2B,C**). Of note, loss of UBR5 had no effect on the half-life of APH-2 protein (data not shown). It is possible there are additional E3 ligases that regulate APH-2 and APH-2 protein stability does not solely depend on UBR5.

Given the difference in HBZ half-life in the presence and absence of UBR5, we examined HBZ ubiquitination using both coIPs (**Figure 4**) and MS/MS analysis (**Figure 5**). Upon ubiquitin enrichment, we identified both HBZ and UBR5 interaction (**Figure 4A**). Likewise, upon HBZ enrichment, we identified several higher molecular weight bands using ubiquitin antibody (**Figure 4B**). The predominant ubiquitin band, indicated with arrow 2, is decreased in UBR5 knockdown cells, indicating UBR5 is responsible for HBZ ubiquitination. The principal ubiquitin bands, indicated with arrow 1 and 2, were subjected to MS/MS analysis and 7 ubiquitinated lysine residues were identified throughout the HBZ protein (K37, K119, K120, K153, K155, K181, K186) (**Figure 5**). A high percentage of the protein (45%) was covered in our analysis, especially the important C-terminal bZIP region. shRNA-mediated knockdown of UBR5 decreased the amount of ubiquitinated HBZ, again confirming UBR5 ubiquitinates HBZ. In a recent report, we found acetylation, phosphorylation, and methylation of the HBZ protein (Dissinger et al., 2014). However, mutational analysis of the modified residues failed to identify any effects on known HBZ functions. Surprisingly, several of the methylated (K37, K181, K186) and acetylated (K155) lysine residues in HBZ were also identified as ubiquitinated. It is possible that methylation or acetylation at these residues may inhibit the deposition of other PTMs, such as ubiquitination (Caron et al., 2005; Lanouette et al., 2014). Therefore, the role of HBZ methylation and acetylation could be to prevent ubiquitination and stabilize HBZ. Conversely, ubiquitination could prevent methylation and/or acetylation of these residues. In addition to promoting degradation, protein ubiquitination can also alter cellular localization, affect protein activity, and promote or prevent other protein interactions. The effect of HBZ ubiquitination on these various activities is of interest and will require further study.

UBR5 has been implicated in several cancers such as lung, breast and ovarian. UBR5 was upregulated in both breast and ovarian cancer due to an allelic imbalance (Clancy et al., 2003). The same report found a survival disadvantage for breast cancer patients with UBR5 dysregulation. A recent study for mantle cell lymphoma showed non-synonymous mutations in UBR5 in roughly 18% of MCL cases (Meissner et al., 2013). Most of these mutations are near the carboxy-terminus of UBR5, which is associated with the E3 ubiquitin ligase function. Data from The Cancer Genome Atlas (TCGA) shows UBR5 amplification as a common alteration in many cancer types^1^. However, the level of UBR5 in T-cell lymphomas was not included. Here, we report an upregulation of UBR5 in T-cell leukemia/lymphoma cell lines (**Figure 6**). Surprisingly, UBR5 mRNA is decreased, suggesting a post-transcriptional method of regulation. Upon further examination, we found a steady increase in UBR5 protein expression during *in vitro* T-cell transformation by HTLV-1. It will be important to follow-up and determine whether UBR5 expression is correlated to disease progression in ATL and HAM/TSP patients, as UBR5 may serve as a diagnostic marker. Recent work has shown HBZ can localize in different subcellular compartments depending upon the pathology associated to HTLV-1 infection (Baratella et al., 2017). Although UBR5 is mainly localized to the nucleoplasm, it can also be localized to the cytosol (The Human Protein Atlas). It will be of interest to determine if UBR5 also affects the stability of HBZ when it is segregated in the cytoplasm.

Recently, UBR5 was found to regulate gastric cancer cell growth both *in vitro* and *in vivo* (Yang et al., 2016). Using shRNA-mediated lentiviral knockdown, we found loss of UBR5 decreased T-cell lymphoma proliferation *in vitro* (**Figure 7A**, Jurkat cells). However, since UBR5 regulates the stability of HBZ, loss of UBR5 resulted in an increase in HBZ and therefore no difference in cell proliferation in HTLV-1-transformed cells (**Figure 7A**, SLB-1 cells). Upon further examination, we found that loss of UBR5 caused an increase in the level of apoptosis in Jurkat cells. Using Jurkat-HBZ cells we were able to rescue this difference due to the anti-apoptotic effects of HBZ (**Figure 7B**).

HBZ and Tax frequently have opposing effects on signaling pathways and various cellular processes. Tax is able to enhance viral transcription, whereas HBZ inhibits Tax-mediated transcription (Gaudray et al., 2002; Lemasson et al., 2007). Tax induces cell senescence whereas HBZ promotes cellular proliferation (Arnold et al., 2006, 2008; Satou et al., 2006; Ho et al., 2012). Tax activates NF-κB signaling whereas HBZ represses p65-mediated signaling (Ballard et al., 1988; Zhao et al., 2009). By having opposing effects, the virus is ensuring homeostasis within the cell. Thus far, the field has focused on regulation of Tax functions without regard for regulation of HBZ protein. Regulation of HBZ protein levels could very well have significant implications on Tax functional effects. A recent report details the occurrence of Tax gene bursts in HTLV-1-infected T-cells from patients (Billman et al., 2017). This result suggests that Tax may indeed be expressed throughout infection and highlights the relevance of HBZ regulation and the Tax/HBZ interplay.

We now show HBZ is ubiquitinated, presumably by the cellular E3 ubiquitin-protein ligase, UBR5. In addition, our work demonstrates the importance of UBR5 on the proliferative phenotype of transformed T-cell lines.

## Author Contributions

AP, PG, and MK conceived and planned the experiments. AP, JA-S, CH, and NS carried out the experiments and analyzed the data. AP took the lead in writing the manuscript. All authors provided the critical feedback and helped to shape the research, analysis, and the manuscript.

## Conflict of Interest Statement

The authors declare that the research was conducted in the absence of any commercial or financial relationships that could be construed as a potential conflict of interest.

## Footnotes

1 http://cancergenome.nih.gov/
